# Formation and Yield of Multi-Walled Carbon Nanotubes Synthesized via Chemical Vapour Deposition Routes Using Different Metal-Based Catalysts of FeCoNiAl, CoNiAl and FeNiAl-LDH

**DOI:** 10.3390/ijms151120254

**Published:** 2014-11-05

**Authors:** Mohd Zobir Hussein, Adila Mohamad Jaafar, Asmah Hj. Yahaya, Mas Jaffri Masarudin, Zulkarnain Zainal

**Affiliations:** 1 Advanced Material and Nanotechnology Laboratory, Institute of Advanced Technology (ITMA), Universiti Putra Malaysia, 43400 UPM Serdang, Selangor, Malaysia; E-Mail: adilamj@upm.edu.my; 2 Department of Chemistry, Faculty of Science, Universiti Putra Malaysia, 43400 UPM Serdang, Selangor, Malaysia; E-Mails: hamsajaya@upm.edu.my (A.H.Y.); zulkar@upm.edu.my (Z.Z.); 3 Department of Cell and Molecular Biology, Faculty of Biotechnology and Biomolecular Sciences, Universiti Putra Malaysia, 43400 UPM Serdang, Selangor, Malaysia; E-Mail: masjaffri@upm.edu.my

**Keywords:** chemical vapor deposition, layered double hydroxides, hexane, MWCNTs

## Abstract

Multi-walled carbon nanotubes (MWCNTs) were prepared via chemical vapor deposition (CVD) using a series of different catalysts, derived from FeCoNiAl, CoNiAl and FeNiAl layered double hydroxides (LDHs). Catalyst-active particles were obtained by calcination of LDHs at 800 °C for 5 h. Nitrogen and hexane were used as the carrier gas and carbon source respectively, for preparation of MWCNTs using CVD methods at 800 °C. MWCNTs were allowed to grow for 30 min on the catalyst spread on an alumina boat in a quartz tube. The materials were subsequently characterized through X-ray diffraction, Fourier transform infrared spectroscopy, surface area analysis, field emission scanning electron microscopy and transmission electron microscopy. It was determined that size and yield of MWCNTs varied depending on the type of LDH catalyst precursor that is used during synthesis. MWCNTs obtained using CoNiAl-LDH as the catalyst precursor showed smaller diameter and higher yield compared to FeCoNiAl and FeNiAl LDHs.

## 1. Introduction

Layered double hydroxides (LDHs), also variedly known as anionic clays, can be structurally described as stacks of positively charged layers intercalated with anions within its inter-spacing. The structure of LDHs follows that of brucite-like layers, in which a divalent metal cation is located within the center of an oxygen octahedra, and two-dimensional infinite layers are formed through edge sharing of the octahedra. The partial isomorphous substitution of trivalent cations for divalent cations results in a net positive charge of the layers. Conferment of a positive charge allows for any organic or inorganic anions to be readily intercalated between the brucite layers, in order to maintain a structural charge balance. Often, water molecules arising from the crystallization process also associates within these interlayer galleries. LDHs classes of materials generally follow a chemical formula representing of [M^II^_1−x_ M^III^_x_ (OH)_2_]^b+^ [A^m−^_b/m_].nH_2_O, where M (II) is a divalent cation, M (III) is a trivalent cation and A is an anion with charge of n [1].

The interlayer spacing of LDH has been shown to provide for a potent reactive environment, even in gentle thermal treatments. Calcination reactions at intermediate temperatures (450–600 °C) showed persistence of the layered brucite, but subsequently collapsed at significantly higher temperatures. Calcined LDH products often exist in the form of mixed metal oxides. During LDH calcination under inert gas environments, both spinel M(II)M(III)_2_O_4_ and free M(II)O are frequently produced. Mixed metal oxides have attracted an appreciable research fascination, both as catalysts and catalyst supports, due to their high metal dispersion, and stable-supported metal particles, which possess both basic and acidic group sites. The mixed oxide catalysts obtained by thermal decomposition of LDHs also confer advantageous modifications of enhanced surface areas, thus creating active sites for reactions to readily occur with the possibility of higher percentage product outputs [1].

Since its discovery by Iijima in 1991, carbon nanotubes (CNT) have garnered great interest in material science research, both from a fundamental perspective, as well as its potential for various practical applications. CNTs are versatile nanosized structures, with unique electronic, mechanical, optical, and chemical characteristics that pave the way towards a myriad of potential interdisciplinary applications. These types of materials have been especially studied for applications in transistors, field-emission tips, sensors, supercapacitors and in the biomedical field [2,3,4,5,6]. Apart from electric-arc discharge and laser ablation techniques, carbon nanotubes can also be prepared through catalytic pyrolysis of carbon-containing gases via catalytic chemical vapor deposition (CCVD) [7]. The CCVD technique has been widely explored in the production of several CNT, such as single-walled, double-walled, and multi-walled derivatives [8,9,10]. Concurrently, efforts have now focused towards the determination of optimal catalysts for efficient nanotube fabrication, which mostly consists of Fe, Co, Ni elements over porous material supports, or high-surface-area oxide matrices that increase their reactivity as catalyst clusters [11,12,13,14].

The successful application of metal oxides catalyst derived from LDH-based materials have attracted its application in the synthesis of CNT formation through carbon vapor deposition routes. Recently, the efficacy of LDHs as catalyst precursors for the synthesis of carbon nanotubes via catalytic chemical vapor deposition of acetylene has been reported. Nanometer-sized cobalt particles were prepared by the calcination and subsequent reduction of a single LDH precursor containing cobalt (II) and aluminum ions homogeneously dispersed at the atomic level. The Co nanoparticles have been employed as catalytically active sites for growth of CNTs. Multi-walled carbon nanotubes (MWCNTs) with uniform diameters were obtained [15]. Enhanced catalytic activities can be observed by incorporating transition metal cations into the brucite-like layers of LDHs, contributed by the unique properties of the final catalysts, such as high metal dispersion and large surface area after a controlled thermal treatment. Better control in the dispersion and size of the catalyst particles is also achieved, through the ordered prearrangement of metal cations in the layers of the LDH precursor at an atomic level.

This study reports the application of three metal-based catalysts; FeCoNiAl-DH, CoNiAl-LDH, and FeNiAl-LDH for use in the formation of CNTs. The preparation of CNTs via use of LDH-based catalysts confers the advantage of low synthesis temperatures using cheap, simple instrumentation, and a robust prospective for large-scale productions. Here, FeCoNiAl-DH, CoNiAl-LDH, and FeNiAl-LDH previously prepared at the fixed ratio of *R* = 4 was initially prepared via co-precipitation methods. The resulting FeCoNiAl, CoNiAl, and FeNiAl composite oxides were then obtained by calcination of corresponding LDH precursors at 800 °C, and were then used as catalyst or substrate in the formation of carbon nanotubes. The influence of these three types of material towards growth of CNTs was then examined and visualized via various means of physiochemical analyses.

## 2. Results and Discussion

### 2.1. Carbon Yield

The catalytic activity of CoNiAl, FeNiAl and FeCoNiAl mixed oxide catalyst were tested in hexane decomposition at a reaction temperature of 800 °C. As expected, different mixed oxide catalysts notably affected carbon yield. Carbon yields of 183.5%, 124.8%, and 110.5% were obtained for synthesized CoNiAl-CNT, FeCoNiAl-CNT and FeNiAl-CNT, respectively.

### 2.2. Powder X-ray Diffraction

**Figure 1:**
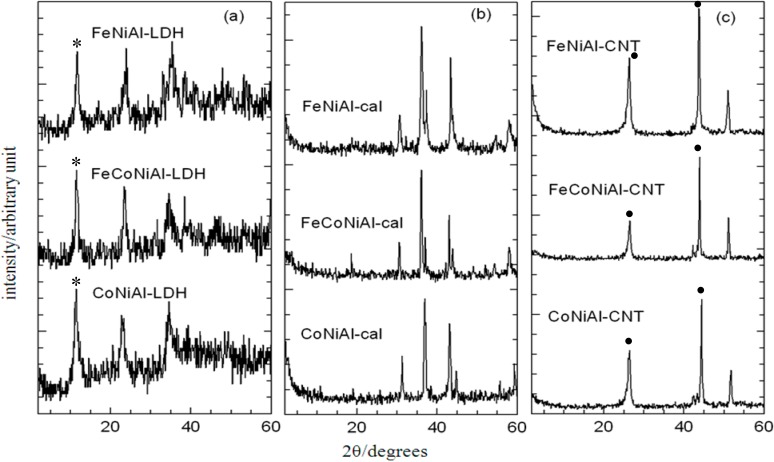
Powder X-ray diffraction (PXRD) patterns of layered double hydroxides (LDH) (**a**) calcined LDH (**b**) and CNT over calcined LDH (**c**). Asterisks (*) show characteristic peaks for LDH at 8.5 Å (003). Dots (•) indicate the characteristic peaks for carbon (26.1 Å, 44.8 Å), indicating the formation of CNT.

Figure 1a shows the x-ray diffraction (XRD) patterns of the catalysts CoNiAl-LDH, FeCoNiAl-LDH, and FeNiAl-LDH precursors. Characteristic reflections corresponding to hydrotalcite-like LDHs were observed in all three samples, indicating a potent formation of brucite structures. Other crystalline phases non-indicative of the LDH structure were not detected. The narrow and sharp reflections observed had suggested that the LDH products confer good crystallinity and structural integrity. However, XRD patterns of LDH samples following calcination (Figure 1b) did not show these characteristic reflections, which indicates an absence of the layered structure of LDHs. This observation was expected, due to the collapse of the LDH metal layers after thermal treatment.

The use of the LDH precursors with uniformly distributed cations seemed to facilitate the formation of spinel phases [16]. However, it is difficult to distinguish the different phases, due to its superposition of characteristic reflections in the XRD spectras. The position and relative intensity of the reflections were basically identical for all samples. Powder x-ray diffraction (PXRD) patterns for synthesized CNT materials are as shown in Figure 1c, where a peak centered at approximately 26.1° in all three samples was determined as the reflection plane of graphite, thus confirming existence of a carbon element [17] that is CNTs.

### 2.3. Fourier Transform Infrared

**Figure 2:**
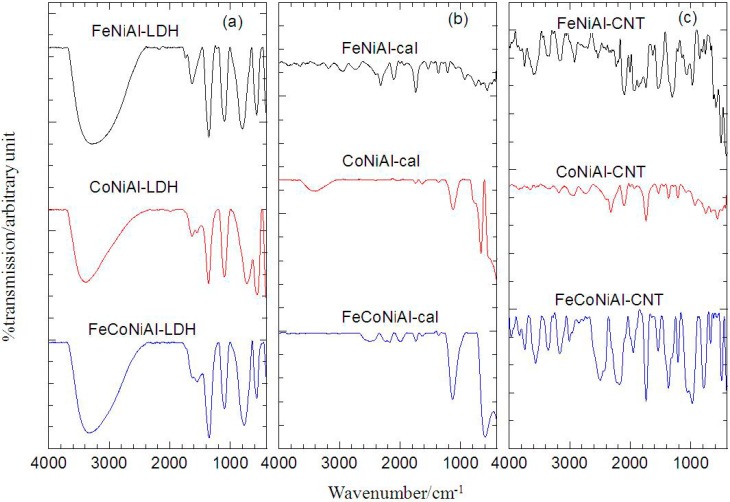
FTIR of LDH (**a**) calcined LDH (**b**) and CNT over calcined LDH (**c**).

The Fourier Transform Infrared (FTIR) spectra in Figure 2a show the typical features of LDH with carbonate as the anion in the interlayer. The FTIR analysis shows that the appearance of a broad band at 3277–3388 cm^−1^ corresponds to the O-H vibration mode, υ_OH_. At around 1630–1634 cm^−1^, weak bands could be observed which is attributed to the δ_H2O_. Strong peaks in the range of 1351–1360 cm^−1^ correspond to the vibration of CO_3_^2−^. Sharp and strong bands located at less than 1000 cm^−1^ correspond to MO vibrations and MOH bending [18,19]. Figure 2b shows that the O-H vibration mode groups at 3409 cm^−1^ had decreased in calcined CoNiAl material, and was absent in other calcined samples. However, this O-H vibration mode peak intensity was still found uncalcined LDH, suggesting a dissociation of the LDH structure in calcined materials. The thermal treatment of LDHs has also shown its effect on the CO_3_^2−^ vibration band which caused the band to decrease in size. As discussed in the FTIR of LDHs, the bands at lower wavenumber are due to the vibrations of M-O, M-O-M, and O-M-O bonds in the layers, which are typical for this kind of layered solids [20]. There are a few infrared active modes of CNTs and it depends on the symmetry of the CNTs, which is chiral, zigzag and armchair [21]. In Figure 2c, the features at 1739–1745 cm^−1^ [22] and 1537–1541 cm^−1^ [23,24] are attributed to the MWCNT vibration modes. From the FTIR analysis, the CNTs were observed to exist in the sample and this is in agreement with the PXRD analysis.

### 2.4. Surface Area Analysis

**Figure 3:**
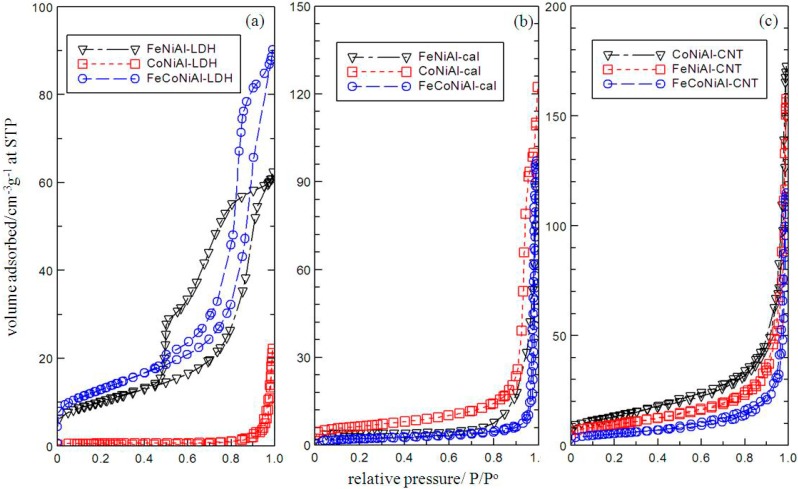
Adsorption-desorption isotherms of LDH (**a**) calcined LDH (**b**) and CNT over calcined LDH (**c**).

**Figure 4:**
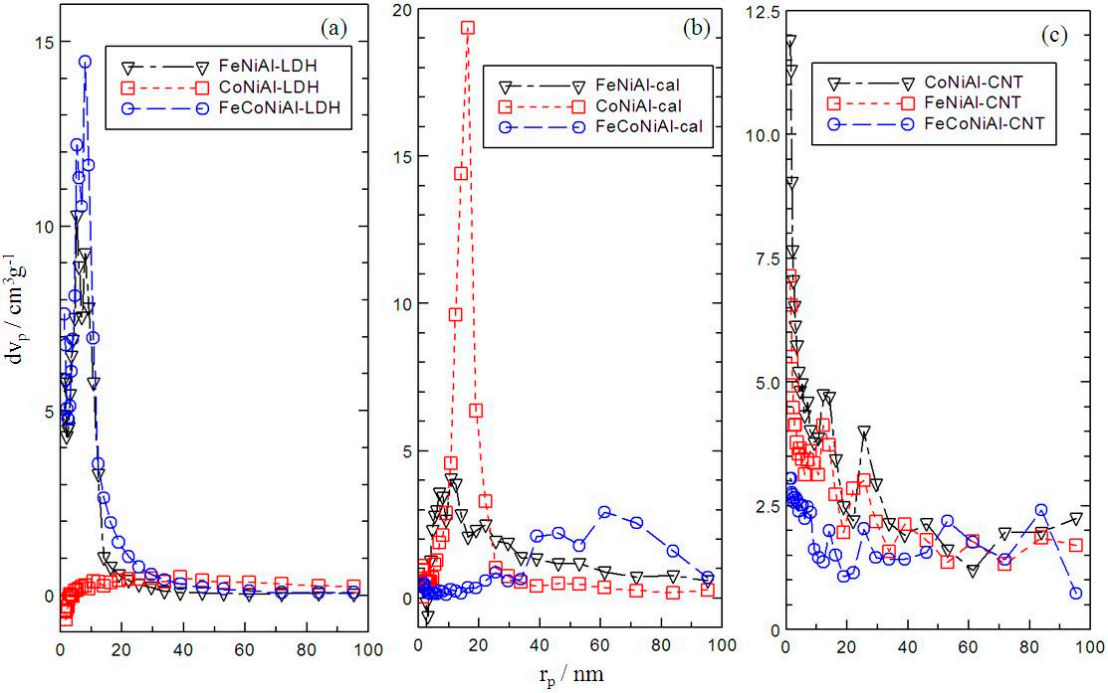
Pore size distribution of LDH (**a**) calcined LDH (**b**) and CNT over calcined LDH (**c**).

From Figure 3, FeCoNiAl, CoNiAl and FeNiAl based material in the form of LDHs (a), calcined LDHs (b) and CNTs (c) exhibited surface properties of Type IV isotherms which can be attributed by the mesoporous-type structure (20–500 Å) [25]. All of the isotherms showed almost the same trend of little difference being exhibited at low relative pressures. However, the slope increase at high relative pressures indicates an increased uptake of adsorbate due to adsorption in mesopores, which leads to multilayer formation until a certain pressure where condensation takes place. A very narrow H3 hysteresis loop could be observed at high pressures, which exhibit no limit to the adsorption at high P/P° [25]. All of the adsorption and desorption branches shown in Figure 3 are parallel except for FeNiAl and FeCoNiAl-LDH which could be due to the complex pore structures. Figure 4a–c showed that the pore was distributed randomly at 1–90 nm in FeCoNiAl, CoNiAl and FeNiAl based material in the form of LDHs, calcined LDHs and CNTs, respectively. Significant modifications in the pore size of CoNiAl based material could be observed as shown in Table 1. CoNiAl-CNT possesses the highest BET surface area at 47.56 m^2^·g^−1^ and the smallest BJH desorption average pore diameter value of 0.98 nm, which could lead to the formation of carbon nanotubes with small diameters.

**Table 1:** Surface properties of FeNiAl, CoNiAl and FeCoNiAl LDH, calcined LDH and CNT over calcined LDH.

Material		BET Surface Area (m^2^·g^−1^)	BJH Desorption Average Pore Diameter (nm)
LDH	FeNiAl	35.1	5.3
	FeCoNiAl	32.5	8.0
	CoNiAl	1.0	39.0
Calcined LDH	FeNiAl	6.4	10.7
	FeCoNiAl	6.7	8.9
	CoNiAl	22.3	1.6
CNT	FeNiAl	33.7	1.2
	FeCoNiAl	19.8	1.6
	CoNiAl	47.6	0.98

### 2.5. Field Emission Scanning Electron Microscope

**Figure 5:**
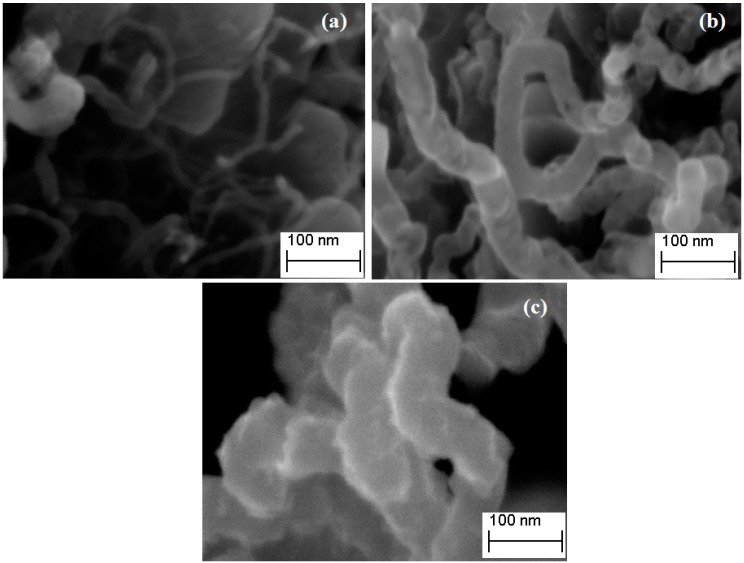
Field Emission Scanning Electron Microscope (FESEM) micrograph of CoNiAl-CNT (**a**); FeNiAl-CNT (**b**) and FeCoNiAl-CNT (**c**).

Figure 5 shows Field Emission Scanning Electron Microscope (FESEM) images of obtained CNTs with various morphologies. As observed in Figure 5a, CoNiAl-CNT was found to be the smallest diameter, followed by FeNiAl-CNT (Figure 5b) and FeCoNiAl-CNT (Figure 5c). Reasons leading to the formation of different sized CNTs using different types of LDH might include the composition of metal interaction in the layers and the distribution of metals when calcination took place. These have an effect on the size of the metal catalyst, which influences the growth of carbon nanotubes [26]. CoNiAl-CNT produced had smooth surfaces, were long and straight, and entangled implying that the synthesized CNTs are of good quality and well graphitized. Both FeNiAl-CNT and FeCoNiAl-CNT display helical nanotubes with rough surface morphology. However, FeCoNiAl-CNT shows entanglement of every part of the nanotubes.

### 2.6. Transmission Electron Microscope

**Figure 6:**
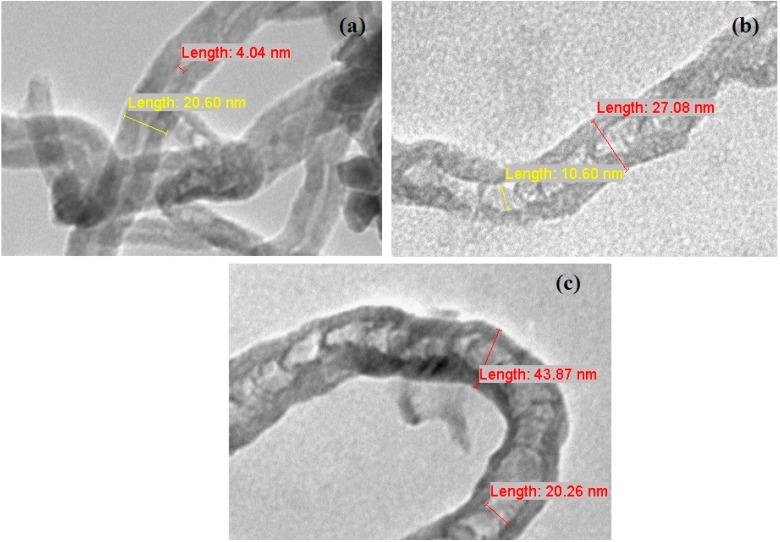
Transmission Electron Microscope (TEM) images of carbon nanotubes of CoNiAl-CNT (**a**); FeNiAl-CNT (**b**) and FeCoNiAl-CNT (**c**).

Figure 6 shows Transmission Electron Microscope (TEM) observations of differences in the structures of CNT when different mixed oxides as catalysts were used. CoNiAl-CNT in Figure 6a exhibited CNTs entangled with each other and contained lower amounts of amorphous carbon on their surfaces, indicating high quality CNTs. Both FeNiAl-CNT (Figure 6b) and FeCoNiAl-CNT (Figure 6c) show defects such as kinks and bends in the tubes. The outer diameters of nanotubes are shown in Figure 6, which exhibit diameters of 20.60, 27.08 and 43.87 nm for CoNiAl-, FeNiAl-CNT and FeCoNiAl-CNT, respectively. As observed, Figure 5 and Figure 6 complement each other. The size of CoNiAl-CNT, which shows the smallest diameter, could be due to the stable active cobalt clusters, impeding agglomeration among other particles and therefore leading to better dispersion of active metal particles, which finally influence the formation of the CNTs [1,27]. Here, it is believed that the difference in the diameters of CNTs grown with different types of metal catalysts is principally attributed to the different agglomeration of existing metallic metal particles.

## 3. Experimental Section

### 3.1. Preparation of LDH Precursors

The LDHs of Co-Ni-Al-SO_4_^2−^ (CoNiAl), Fe-Ni-Al-SO_4_^2−^ (FeNiAl), and Fe-Co-Ni-Al-SO_4_^2−^ (FeCoNiAl) were synthesized through co-precipitation methods. The molar ratios of cobalt:aluminium, nickel:aluminium, and iron:aluminium were set at 4:1. Mixed aqueous solutions of cobalt, nickel, iron, and aluminium nitrates were prepared at pH 10.00 ± 0.05 by dropwise addition of aqueous NaOH solution (2.00 M) with vigorous stirring. The titration of NaOH was performed under the constant flow of nitrogen gas to avoid, or at least minimize contamination by atmospheric CO_2_ throughout the experiment. The precipitate was then aged at 70 °C for 18 h, washed, and dried in the oven at 70 °C. The dried samples were then ground into fine powder by a mortar and pestle, before kept in sample bottles pending further use and characterisation. The as-synthesized LDH samples were calcined in air at 800 °C for 5 h at a heating rate of 4 °C/min. The resulting mixed metal oxides were then slowly cooled to room temperature.

### 3.2. Growth of CNTs

CNTs were synthesized by catalytic chemical vapor deposition of hexane, in a quartz tube housed inside a horizontal tube furnace equipped with gas flow controller and temperature-programmed control. After loading the calcined LDH samples in an alumina boat, the temperature in the furnace was raised from room temperature to 800 °C at a rate of 4 °C/min, under nitrogen gas flow at 50 PSIG for 150 min. After 120 min hexane was introduced, and the temperature was maintained for a subsequent 30 min before the whole system was turned off. The furnace was then left to cool to room temperature. The resulting synthesized CNTs were kept in sample bottles pending further use and characterisation. For calculation of percentage carbon yield, the following equation was used;

$$\text{Carbon yield (\%)}=\frac{\text{mass of carbon deposited onto the catalyst × 100}}{\text{initial mass of mixed oxide}}$$

### 3.3. Characterisation

Powder X-ray diffraction (PXRD) patterns of the samples were collected using an ITAL Structure APD 2000 instrument. The CuKα used was at the wavelength λ = 0.1540562 nm and scanning rate was set at 2 degrees·min^−1^. Infrared absorption spectras of each sample were analyzed in a FTIR spectrophotometer in the form of KBr pellets, using a Perkin-Elmer model 1725X, in the wavelength range of 400–4000 cm^−1^. The N_2_ adsorption-desorption isotherm, the specific surface area and pore size distribution was carried out by using a BELSORP-mini. Before the analysis, the samples were degassed at 105 °C under vacuum environment. CARL ZEISS SUPRA 40VP operated at 5 kV was used for field emission scanning electron microanalyses (FESEM) of the samples. Transmission electron microscopy (TEM) images were taken using Hitachi H-7100 operated at 40 kV.

## 4. Conclusions

Carbon nanotubes were grown on a series of catalysts derived from CoNiAl-LDH, FeCoNiAl-LDH and FeNiAl-LDH materials. The catalytically active Co, Ni and Fe species for MWNTs growth were successfully formed by calcination of LDHs at 800 °C. Different mixed oxides catalyst precursors were found to produce different carbon yields of CNTs, as well as different sizes and structure of the formed MWNTs. CoNiAl mixed oxide was found to give the highest yield of CNTs with less amorphous carbons on their wall surfaces, indicating high quality CNTs were produced.
